# Anaphylaxis due to sulfite intolerance: a protective effect from cyanocobalamin

**DOI:** 10.1186/2045-7022-3-S3-P15

**Published:** 2013-07-25

**Authors:** PM Ojeda, I Ojeda, G Rubio

**Affiliations:** 1 Allergy, Clínica de Asma y Alergia Dres. Ojeda, Madrid, Spain

## Background

The mechanisms of sulfite intolerance are unknown. It has been suggested that, in some cases, it could be due to a partial sulfite-oxidase deficiency. In these patients, this enzyme is unable to metabolize adequately an overload of ingested or inhaled exogenous sulfite, triggering a bronchospasm. Cyanocobalamin is able to catalyze the extracellular oxidation of sulfites, preventing sulfite accumulation and the subsequent symptoms due to this excess.

## Methods

A 36 y.o. woman was attended at our allergy Clinic after suffering repeated anaphylactic episodes and an anaphylactic shock, 60 minutes after eating different foods. The allergic study confirmed sensitization to pollens, animal dander, dust mites, molds and PR-10 proteins (ImmunoCAP Isac, Thermofisher, Sweden). We performed a double-blind placebo controlled challenge (DBPCC) with sulfites. She reacted with 50 mg of metabisulfite with facial flushing, rhinitis and bronchospasm (FEV1 decrease > 20%).

We recommended a sulfite avoidance diet, but two months later she came back complaining of great difficulty to avoid sulfite given the ubiquity of this additive in nearly all foods.

We performed another sulfite challenge with cyanocobalamin pretreatment.

## Results

We pre-treated the patient with 4 mcg of cyanocobalamin 1 hour before repeating de sulfite challenge. We increased the sulfite dose every 20 minutes, from 5 mg to 200 mg. The challenge test was negative. We recommended the patient to take every day 4 mcg of cyanocobalamin in order to prevent anaphylactic reactions from inadvertent exposure to sulfites and to continue with a sulfite avoidance diet. Since then, she has been stable, without asthma exacerbation or anaphylactic reactions.

## Conclusion

We describe a case of sulfite intolerance with anaphylactic reactions, in which we have been able to demonstrate a protective effect from cyanocobalamin.

## Disclosure of interest

None declared.
